# Implementation, mechanisms of change and contextual factors of a complex intervention to improve interprofessional collaboration and the quality of medical care for nursing home residents: study protocol of the process evaluation of the *interprof* ACT intervention package

**DOI:** 10.1186/s13063-022-06476-6

**Published:** 2022-07-08

**Authors:** Linda Steyer, Christian Kortkamp, Christiane Müller, Britta Tetzlaff, Nina Fleischmann, Clarissa E. Weber, Martin Scherer, Anja Kühn, Anne-Marei Jarchow, Frederike Lüth, Sascha Köpke, Tim Friede, Hans-Helmut König, Eva Hummers, Indre Maurer, Katrin Balzer

**Affiliations:** 1 Institute for Social Medicine and Epidemiology, Nursing Research Group, University of Lübeck, Ratzeburger Allee 160, Haus 50, 23538 Lübeck, Germany; 2 Chair of Organization and Corporate Development, Faculty of Economic Sciences, Georg-August-University Göttingen, Platz der Göttinger Sieben 3, 37073 Göttingen, Germany; 3 Department of General Practice, University Medical Center Göttingen, Humboldtallee 38, 37073 Göttingen, Germany; 4 Department of General Practice and Primary Care, University Medical Center Hamburg-Eppendorf, Martinistraße 52, 20246 Hamburg, Germany; 5 Institute of Nursing Science, University of Cologne, Medical Faculty & University Hospital Cologne, Gleueler Straße 176-178, 50935 Köln, Germany; 6 Department of Medical Statistics, University Medical Center Göttingen, Humboldtallee 32, 37073 Göttingen, Germany; 7 Department of Health Economics and Health Services Research, University Medical Center Hamburg-Eppendorf, Martinistraße 52, 20246 Hamburg, Germany

**Keywords:** Process evaluation, Nursing homes, Interprofessional collaboration, Mixed methods

## Abstract

**Background:**

To improve interprofessional collaboration between registered nurses (RNs) and general practitioners (GPs) for nursing home residents (NHRs), the *interprof* ACT intervention package was developed. This complex intervention includes six components (e.g., shared goal setting, standardized procedures for GPs’ nursing home visits) that can be locally adapted. The cluster-randomized *interprof* ACT trial evaluates the effects of this intervention on the cumulative incidence of hospital admissions (primary outcome) and secondary outcomes (e.g., length of hospital stays, utilization of emergency care services, and quality of life) within 12 months. It also includes a process evaluation which is subject of this protocol. The objectives of this evaluation are to assess the implementation of the *interprof* ACT intervention package and downstream effects on nurse–physician collaboration as well as preconditions and prospects for successive implementation into routine care.

**Methods:**

This study uses a mixed methods triangulation design involving all 34 participating nursing homes (clusters). The quantitative part comprises paper-based surveys among RNs, GPs, NHRs, and nursing home directors at baseline and 12 months. In the intervention group (17 clusters), data on the implementation of preplanned implementation strategies (training and supervision of nominated IPAVs, interprofessional kick-off meetings) and local implementation activities will be recorded. Major outcome domains are the dose, reach and fidelity of the implementation of the intervention package, changes in interprofessional collaboration, and contextual factors. The qualitative part will be conducted in a subsample of 8 nursing homes (4 per study group) and includes repeated non-participating observations and semistructured interviews on the interaction between involved health professionals and their work processes. Quantitative and qualitative data will be descriptively analyzed and then triangulated by means of joint displays and mixed methods informed regression models.

**Discussion:**

By integrating a variety of qualitative and quantitative data sources, this process evaluation will allow comprehensive assessment of the implementation of the *interprof* ACT intervention package, the changes induced in interprofessional collaboration, and the influence of contextual factors. These data will reveal expected and unexpected changes in the procedures of interprofessional care delivery and thus facilitate accurate conclusions for the further design of routine care services for NHRs.

**Trial registration:**

ClinicalTrials.gov NCT03426475. Registered on 07/02/2018.

**Supplementary Information:**

The online version contains supplementary material available at 10.1186/s13063-022-06476-6.

## Background

For nursing home residents (NHRs) in developed countries, relatively large rates of emergency and hospital admissions are reported, although the benefits of these admissions for the health and well-being of this population are uncertain [1]. In these countries, the 12-month incidence of hospital admission for NHRs varies from 12 to over 50% [2, 3], with a mean number of 0.3 to 1.3 admissions per NHR per year [2–4]. Analyses suggest that up to half of admissions may be avoidable without posing additional risks to residents’ health and well-being [5–7]. Effective strategies to prevent unnecessary admissions are therefore highly required.

In Germany, as in other countries (e.g., Switzerland, UK, USA), medical care for NHRs is provided by general practitioners (GPs) and other medical specialists who run their own offices or are employed by larger offices or ambulatory medical care centers. Because NHRs are frequently not able to visit a GP’s office, GPs visit nursing homes for regular consultations and are accessible in urgent situations during usual working hours on weekdays. Outside these hours, the NHR and the nursing home staff must draw on out-of-hours GP services and other emergency services when they consider medical support to be necessary. The choice of the GP and other physicians is, by law, at the discretion of the individual NHR. Therefore, nursing homes usually collaborate with a variety of GPs and other medical specialists. Empirical data suggest that on average, ten GPs per nursing home are involved in medical care for NHRs in Germany [8].

A potentially effective strategy to avoid hospitalization is to improve interprofessional collaboration between nursing staff in nursing homes and GPs. Descriptive studies suggest that a continuous flow of information between registered nurses (RNs) and GPs can help prevent hospital admissions [9]. Furthermore, a multicenter qualitative study involving NHRs, their relatives, RNs, and GPs of 18 nursing homes in Germany revealed a need for improvements in shared medical care by GPs and nursing home staff for NHRs, especially with regard to the mutual accessibility of GPs and RNs, person-centered allocation of tasks, scheduling and conduct of GPs’ nursing home visits, and the quality of interprofessional communication and flow of information [10]. Based on these findings, a multicomponent strategy, the “*interprof* ACT intervention package,” was developed and piloted to improve the quality of RN-GP collaboration and thus the quality of medical care for NHRs in Germany [10]. The core components of this intervention package are (1) name badges for GPs and RNs during GPs’ visits, (2) designating a contact person in the GP’s office and among the nursing home’s RNs, (3) a shared definition of mandatory rules for GPs’ availability, (4) standardized procedures for the scheduling, conduct and postprocessing of GPs’ visits in the nursing homes, (5) standardized forms for pro re nata medication prescriptions, and (6) shared goal setting involving the NHR and her/his relative(s), the GP and the RN in charge of the NHR. These components address key elements of interprofessional collaboration and communication in primary care for the elderly [11] and can be adjusted to local requirements and needs, such as pre-existing standards for GPs’ visits or forms for recording pro re nata medication prescriptions (Additional file 1).

**Fig. 1:**
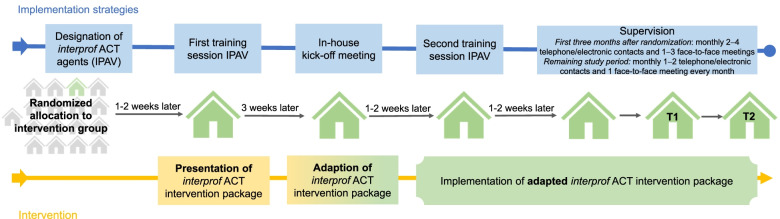
Overview of *interprof* ACT implementation strategies

**Fig. 2:**
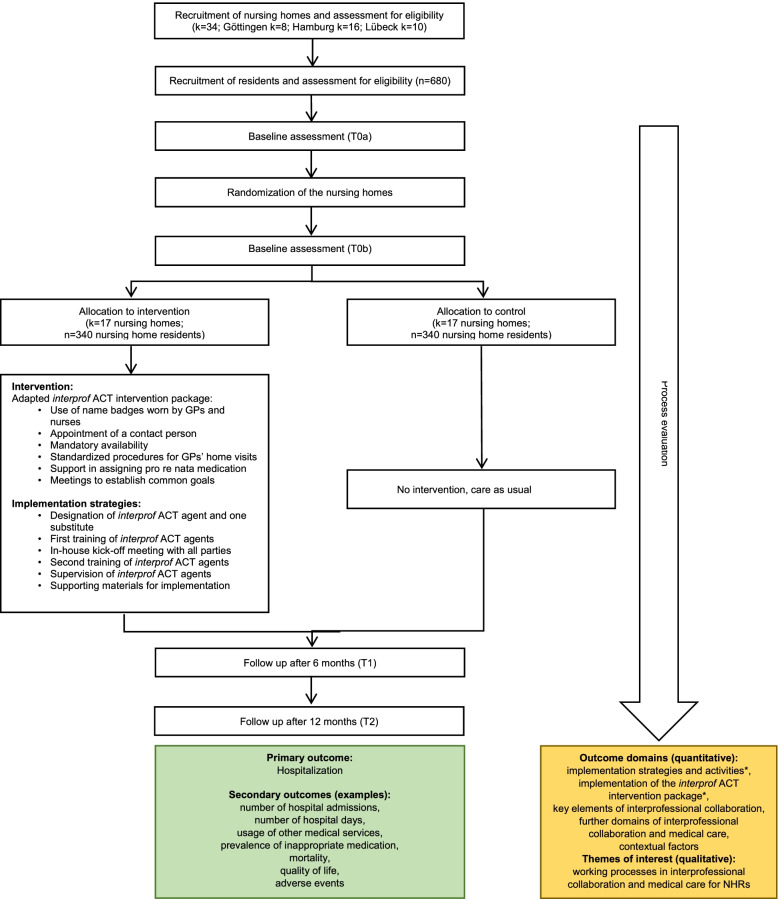
RCT enrollment, group allocation, and follow-ups. *Dose, reach, fidelity, and adaptions. Abbreviations: *GPs* general practitioners, *NHRs* nursing home residents

The *interprof ACT* intervention package is currently being evaluated in a multicenter cluster-randomized controlled trial (CRT) (ClinicalTrials.gov NCT03426475). In this trial, 34 nursing homes across three study centers in Germany (Göttingen, Hamburg, and Lübeck) are randomly assigned to the implementation of the *interprof* ACT intervention package (intervention group) or usual care (control group). In the intervention group, implementation of the intervention package is facilitated by various strategies that aim to involve all local target groups of the *interprof* ACT intervention from the beginning onward. The main strategies are (1) the nomination and training of *interprof* ACT agents (“*interprof* ACT-Verantwortliche”, IPAVs) within the nursing homes, whose main tasks are to champion, coordinate, and monitor the implementation within the facility and maintain close contact with participating GPs and the local study team, (2) an in-house kick-off meeting involving all stakeholders, such as NHRs and their relatives, RNs, GPs, nursing home management, and home advisory boards, to discuss and agree on local adjustments to the *interprof* ACT intervention package, and (3) regular supervision of the IPAVs by the local study team (Fig. 1 and Additional file 1). The main goal of the *interprof* ACT trial is to evaluate the effects of the intervention package concerning the rate of hospital admissions (primary outcome) and other patient-important outcomes, such as quality of life, the utilization of emergency services, or prescriptions of potentially inappropriate medication among participating NHRs within 12 months. Trial outcome data are assessed at baseline (T0), 6 months (T1), and 12 months (T2) post randomization (Fig. 2). The full trial protocol has been published elsewhere [12].

Given the multiple intervention components and the allowed degree of tailoring, the *interprof* ACT intervention package should be viewed as a complex intervention [13, 14]. This complexity is enhanced by various contextual factors at the health system, organizational, and individual levels that may influence the reach, dose, and fidelity of the implementation, including attitudes of health professionals and NHRs as well as the existing local infrastructure and procedures of nurse–physician collaboration and communication [15]. To capture the assumed heterogeneity of the implementation of the *interprof* ACT intervention package, the impact of this implementation on the quality of the RN-GP collaboration, and potential influences of contextual factors, a detailed process evaluation is needed in addition to the main trial [16, 17]. Therefore, a mixed methods process evaluation is embedded into the main trial to achieve systematic insights into the implementation of the *interprof* ACT intervention package (Fig. 2).

**Fig. 3:**
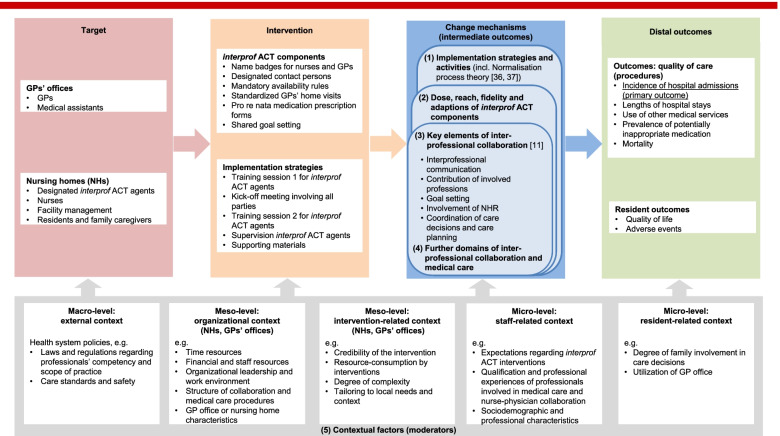
Logic model of the *interprof* ACT intervention package. Numbered bold subheadings represent major outcome domains of the process evaluation. Abbreviation: *GPs *general practitioners

The process evaluation is based on a logic model [18] that illustrates the expected causal pathway of the *interprof* ACT intervention and potential moderating effects of relevant contextual factors [15]. Within this model, it is assumed that the implementation strategies described above induce various activities by the IPAV, the nursing staff, and GPs to adopt and integrate the jointly chosen *interprof* ACT components into their daily routines. The degree and quality of this implementation, as measured by the dose and reach or the fidelity of implemented components, ultimately affect key elements of the RN-GP collaboration. These key elements are based on the conceptual model of interprofessional collaboration in primary elderly care [11] and are expected to mediate the effects of the *interprof* ACT intervention on the distal outcomes, i.e., the primary and secondary patient-important outcomes of the main trial (Fig. 3).

## Methods

### Aims

The overall aims are to assess the change in RN-GP collaboration induced by the implementation of the *interprof* ACT intervention package and to identify contextual determinants of implementation success and its downstream effects on intermediate outcomes. Following existing frameworks for process evaluations of complex interventions [16, 17], this overall aim is divided into several sub-objectives, each focusing on one specific part of the assumed causal pathway from group allocation to the impact of the interventions on the intermediate outcomes (i.e., the interprofessional collaboration). To capture the whole picture, the process evaluation comprises a quantitative part and a qualitative part. The main objective of the quantitative part is to estimate the degree of implementation of the *interprof* ACT components and its downstream effects on the quality of the RN-GP collaboration. Therefore, it addresses the following research questions:

- What is the degree of implementation of the *interprof* ACT components with regard to dose and reach, fidelity, and local adaptations? To what degree does this implementation output vary between clusters?
- What are the effects of the implementation of *interprof* ACT components on the quality of RN-GP collaboration?
- Which contextual factors at the system, organization, intervention, staff, and NHR levels influence the implementation of the *interprof* ACT components as well as the quality of RN-GP collaboration?

The qualitative part mainly aims to assess the *interprof* ACT intervention with regard to feasibility and prospects for successive implementation into routine care. Therefore, it mainly addresses the following research questions:

- How do relevant working processes change as a result of the implementation of *interprof* ACT intervention package?
- How can effective processes be described and defined in a standardized form (process map)?

Altogether, the results of the process evaluation will facilitate the understanding of causal mechanisms behind the main trial findings and point to important facilitators and barriers as well as preferred strategies for large-scale implementation of the *interprof* ACT intervention package in routine care given that the trial results will be in favor of the intervention.

### Design

This study uses a mixed methods triangulation design [19] that combines the collection and analysis of quantitative data on the process, context, and output (nurse–physician collaboration, medical delivery) of the intervention implementation with concurrent qualitative in-depth inquiries of the implementation procedures and context-bound influences. Data for the quantitative part will be repeatedly collected alongside the trial in all trial clusters by means of questionnaires, standardized interviews, and minutes targeting all parties involved in the intervention package implementation and/or the medical care for NHRs. The qualitative data collection will be conducted in a subsample of eight nursing homes and mainly focuses on the interaction between involved health professionals and their working processes. Data will be collected by a combination of non-participating observations and semistructured interviews conducted at different measurement points throughout the trial (Table 1).

**Table 1:** Overview of data sources and measuring points by target groups for process evaluation (quantitative/qualitative)

Target population	Data collection	Measurement points							Target population and recruitment strategies	Sample size
		T0a	T0b	First training (only in IG)	Kick-off meeting (only in IG)	Second training (only in IG)	T1	T2		
*General practitioners (both study groups)*	Quantitative	x (Q)			x^a^ (Q)			x (Q)	All GPs involved in the medical care for participating NHRs and interested in participation For recruitment: • GPs will be invited in written form to take part in the *interprof* ACT trial, including. the process evaluation • All GPs will receive an expense allowance.	Data from a previous trial conducted in the German long-term care setting suggest a resident-to-physician ratio of approximately 3. A total number of about 210 GPs to be invited to participate in the process evaluation will be expected [8]. Based on previous data, a response rate of 45% is assumed, reducing the sample size to about 95 GPs [20–22].
	Qualitative		x (I, O)		x (O)		x (I, O)	x (I, O)	GPs who participate in the main trial and agree to participate in the process evaluation. For recruitment: GPs will be contacted in writing and/or via telephone by members of the research team.	On average, two GPs per NH per measurement point are expected. Participation at each kick-off meeting (*n* = 5) of the subsample of the qualitative process evaluation will be expected.
*Registered nurses (both study groups)*	Quantitative	x (Q)			x^a^ (Q)			x (Q)	All RNs working in direct nursing and medical care for the NHRs throughout the day, evening, or night shifts at data collection days. For recruitment: RNs will be contacted in writing and orally by study team members involved in data collection for main trial outcomes.	On average four RNs may be reached per cluster, representing approximately 30% of the target population [8].
	Qualitative		x (I, O)		x (O)		x (I, O)	x (I, O)	RNs pre-selected by nursing or facility directors based on their involvement in interprofessional collaboration. RNs are included if they are available and willing to take part in the qualitative process evaluation. For recruitment: RNs will be contacted in writing and/or via telephone by members of the research team.	On average, two RNs per NH per measurement point will be expected. Participation at each kick-off meeting (*n* = 5) of the subsample of the qualitative process evaluation will be expected.
*Nursing home residents (both study groups)*	Quantitative	x^b^ (Q)			x^a^ (Q)			x^b^ (Q)	All NHRs participating in the *interprof* ACT trial, i.e., meeting following inclusion criteria [12]: • Have experienced at least one GP contact in the last three months or two GP contacts in the last 6 months or were admitted to the nursing home during the preceding 6 months independently of the actual number of documented GP contacts • Are aged 18 years or older • Provide written informed consent for the study, either by themselves or through their legal guardian Of these, only NHRs able to answer simple questions of satisfaction with medical care by themselves are to be included in the process evaluation. For recruitment: Detailed recruitment strategies and methods to appraise NHRs’ ability to answer questions are described in the main trial protocol [12].	As estimated for the *interprof* ACT trial [12].
	Qualitative				x (O)				NHRs who are co-subject of non-participating observations. For recruitment: In these cases, NHRs will be informed about the observation in written and oral form before the observation takes place. NHRs or their legal guardian must provide informed consent.	Not applicable
*Nursing/ facility directors (both study groups)*	Quantitative	x^c^ (Q)			x^a^ (Q)			x^c^ (Q)	Nursing or facility directors of all nursing homes participating *interprof* ACT trial [12]. For recruitment: Detailed recruitment strategies are described in the main trial protocol [12]. All nursing homes will receive an expense allowance.	As estimated for the *interprof* ACT trial [12].
	Qualitative				x (O)				Only nursing or facility directors who are co-subject of non-participating observations (during kick-off meetings). No specific recruitment strategies are required.	Not applicable
*IPAVs (only in IG)*	Quantitative			x (Q)	x^a^ (Q)	x (Q)	x (Q)	x (Q)	All designated IPAVs or their substitutes. No specific recruitment strategies are required.	In each participating nursing home of the intervention group (*n* = 17), one IPAV and one substitute should be designated, resulting in an expected sample size of approximately *n* = 34.
	Qualitative				x (O)				IPAVs who are: • Involved in direct nursing care and interaction with GPs during non-participating observations, can also be subject of these observations and interviews. • Co-subject of non-participating observations (during kick-off meetings). No specific recruitment strategies are required.	Not applicable
*Study team members (only in IG)*	Quantitative			x (Q, M)	x (Q^a^, M)	x (Q, M)	x^d^ (Q, M)	x^d^ (Q, M)	Involved in the supervision and training of IPAVs No specific recruitment strategies are required.	Approximately 2–4 study team members per study site, resulting in an expected sample size of approximately *n* = 6–12.
	Qualitative				x (O)				Co-subject of non-participating observations during kick-off meetings	Not applicable
*Participants and moderators of the kick-off meetings*	Quantitative				x^a^ (Q)				All relevant stakeholders, especially NHRs and their relatives, RNs, the nursing or facility director of the nursing home, the GPs associated with this nursing home, and the nursing home advisory board. Kick-off meetings will be jointly moderated by the IPAV and one study team member For recruitment: Target persons of the kick-off meetings will be invited orally and in writing by IPAVs of intervention nursing homes.	10 to 15 participants will be expected for each meeting, with one meeting conducted in each of the 17 intervention nursing homes.
	Qualitative				x (O)					

*Abbreviations*: *GP* general practitioner, *I* interview, *IG* intervention group, *IPAV interprof* ACT agent, *M* minutes, *NHR* nursing home resident, *O* observation, *Q* questionnaire, *RN* registered nurse, *T0a* baseline assessment (before randomized allocation), *T0b* shortly post randomization, *T1* follow-up after 6 months, *T2* follow-up after 12 months

^a^Pre-post questionnaire. ^b^In the form of face-to-face interview. ^c^Integrated in case report form of main study. ^d^During supervision (questionnaire for baseline characteristics)

The protocol of this study follows existing recommendations for process evaluations of complex interventions [16, 17] and the reporting of complex interventions [23] and implementation studies (StaRI) [24]. A detailed description of the *interprof* ACT intervention package and preplanned implementation strategies based on these reporting standards is shown in the supplementary data (Additional file 1).

Different trial partners lead the quantitative and qualitative parts. The quantitative part is mainly planned and overseen by the Nursing Research Unit, University of Lübeck, and all three study centers (Departments of General Practice and Primary Care in Göttingen and Hamburg, Nursing Research Unit in Lübeck) are involved in the data collection. The qualitative part is planned and will be conducted by the Chair of Organization and Corporate Development, Faculty of Economic Sciences, Georg-August-University Göttingen. However, both study parts were conceptualized in close collaboration with all trial partners, and the quantitative and qualitative data are assumed to complement each other and are triangulated at several stages of the research process. During the implementation of the trial, preliminary findings from the qualitative inquiries at the first measurement point (T0) will be taken into account in the choice of variables to be included in the quantitative measurements at T1 and T2. At the analysis stage, descriptive findings of both parts will be cross-mapped to inform statistical models to evaluate the downstream effects of the *interprof* ACT intervention on nurse–physician collaboration and the medical care provided to the NHRs.

### Target populations and data sources

The quantitative part of the process evaluation will be conducted in all 34 participating nursing homes, whereas the qualitative part will be performed in a subsample of eight participating facilities (four per study group) with at least one nursing home per study group per study center. For the recruitment of this subsample, local study centers will invite participating nursing homes to take part in this qualitative inquiry until the subsample is completed. Additional criteria, such as the size and location of the nursing homes, will be considered in the recruitment to achieve a heterogeneous sample that allows for the identification of differences arising from these characteristics. Participating nursing homes will receive detailed information about the process evaluation and the quantitative and qualitative data collection methods. Participation in the process evaluation will be voluntary.

Table 1 provides an overview of the populations targeted by this process evaluation at the several measuring points, including information about the data sources used for the quantitative and qualitative parts. Both the quantitative and qualitative parts target RNs and GPs of participating nursing homes (all facilities or subsamples). The quantitative part additionally includes IPAVs, NHRs, nursing home directors, and study team members (Table 1). In the qualitative part, the latter three groups will only be involved when representatives of these groups take part in the kick-off meetings that will be held by nursing homes assigned to the intervention group to discuss the *interprof* ACT intervention and to reach consensus about which components should be implemented in this facility and which local adjustments are required to these components (Additional file 1). The detailed eligibility criteria for each target population and the recruitment strategies are outlined in Table 1. For NHRs, the same eligibility criteria will be applied as in the main trial [12], with the restriction that only residents who are themselves able to answer simple questions about satisfaction with medical care will be eligible.

Table 1 also provides information about the target sample size for each sub-population in the quantitative and qualitative parts of the process evaluation. For the quantitative part, no formal sample size estimation was carried out since this part of the study, like the qualitative part, is explorative by nature. However, the recruitment strategies in place are assumed to yield representative samples of each sub-population.

### Data collection methods

#### Quantitative process evaluation

Table 2 provides an overview of the outcomes measured for the quantitative part of the process evaluation, including the populations targeted by these measurements. The outcomes were chosen based on the logic model underlying this study (Fig. 3). Therefore, data on five major outcome domains will be collected (highlighted in bold numbers in Fig. 3): (1) implementation strategies and activities, (2) implementation of the *interprof* ACT intervention package (as a whole and single components), (3) key elements of interprofessional collaboration, (4) further domains related to interprofessional collaboration and medical care, and (5) contextual factors. The major outcome domains (1) to (4) relate to the assumed change mechanisms (intermediate outcomes), while the major outcome domain (5) reflects potentially relevant moderators. Each of these major domains comprises multiple domains and, in some cases, additional subdomains, which are measured in terms of different dimensions such as attitudes, dose and reach, adaptations, current practice, or self-reported quality and satisfaction.

**Table 2:** Overview of outcome domains and subdomains of the quantitative process evaluation

Domains	Subdomains	Dimensions	Target population and measurement points						Measurement instruments (existing tools used^**a**^)
			IPAVs	RNs	NHDs	NHRs	GPs	STM	
**Implementation strategies and activities**									
Implementation strategies	Kick-off meeting	Attitudes, quality, and satisfaction	(T0b)	(T0b)	(T0b)	(T0b)	(T0b)	(T0b)	Questionnaires, minutes
	IPAV characteristics	E.g., roles and self-efficacy	T0b	–	–	–	–	T0b	Questionnaires, minutes
	IPAV training	Duration, adaptations, quality, and satisfaction	T0b	–	–	–	–	T0b	Questionnaires, minutes
	IPAV supervision	Quality and satisfaction	T0b	–	–	–	–	T0b	Questionnaires, minutes
IPAV work	Collaboration IPAV and IPAV substitutes	Quality and satisfaction	T1, T2	–	–	–	–	–	Questionnaires
	IPAV activities, e.g., in-house training courses and discussions with target groups	Current practice	T1, T2	–	–	–	–	T1, T2	Questionnaires
Implementational work within team (normalization process theory)	Coherence, cognitive participation, collective action, reflexive monitoring	–	T1, T2	T2	–	–	–	–	Questionnaires (NoMAD questionnaire)
***interprof*** **ACT intervention package**									
Intervention package as a whole	–	Dose and reach	T1, T2	–	–	–	–	–	Questionnaires (NoMAD questionnaire), minutes
Name badges		Choice and adaptation, dose and reach, local policies, current practice	T1, T2	T0a, T2	T0a, T2	–	T0a, T2	T1, T2	CRF, questionnaire, minutes
Mandatory availability rules	–	Choice and adaptation, dose and reach, attitudes, current practice, quality, and satisfaction	T1, T2	T0a, T2	T0a, T2	–	T0a, T2	T1, T2	CRF, questionnaire, minutes (Koverdem, InDemA)
Designated contact persons		Choice and adaptation, dose and reach, attitudes, local policies, current practice, quality and satisfaction	T1, T2	T0a, T2	T0a, T2	–	T0a, T2	T1, T2	CRF, questionnaire, minutes (InDemA)
Standardized home visits		Choice and adaptation, dose and reach, attitudes, current practice, quality, and satisfaction	T1, T2	T0a, T2	T0a, T2	T0a, T2	T0a, T2	T1, T2	CRF, questionnaire, minutes (InDemA)
Pro re nata medication		Choice and adaptation, dose and reach, current practice, quality and satisfaction	T1, T2	T0a, T2	T0a, T2	–	T0a, T2	T1, T2	CRF, questionnaires, minutes
Shared goal setting		Choice and adaptation, dose and reach, attitudes, current practice, quality, and satisfaction	T1, T2	T0a, T2	T0a, T2	–	T0a, T2	T1, T2	CRF, questionnaire, minutes (InDemA, Koverdem, PSAT)
**Key elements of interprofessional collaboration**									
Interprofessional communication		Attitudes, current practice, quality, and satisfaction	T1, T2	T0a, T2	T0a, T2	–	T0a, T2	–	CRF, questionnaires (CPAT, Koverdem)
Involvement of NHR		Current practice, quality, and satisfaction	T2	T0a, T2	T0a, T2	T0a, T2	T0a, T2	–	CRF, questionnaires (ZAP)
Contribution of involved professions		Attitudes, current practice, quality, and satisfaction	T1, T2	T0a, T2	–	–	T0a, T2	–	Questionnaires (CPAT, InDemA, Jefferson Scale, PSAT)
Coordination of care decision and care planning		Attitudes, current practice	–	T0a, T2	T0a, T2	–	T0a, T2	–	CRF, questionnaires (InDemA)
**Further domains related to interprofessional collaboration and medical care**									
General interprofessional collaboration		Attitudes, quality and satisfaction, time resources	T1, T2	T0a, T2	T0a, T2	–	T0a, T2	–	CRF, questionnaires (InDemA, Koverdem)
General (medical) care for NHR		Quality and satisfaction utilization of emergency or hospital services (self-reported changes), adverse outcomes	T2	T2	T2	T0a, T2	T2	–	CRF, questionnaires (ZAP, EUROPEP)
Further outcomes		Staff outcome, marketing outcome	–	–	T0a, T2	–	–	–	CRF
**Contextual factors**									
Meso: organizational level	Time resources		T1, T2	–	–	–	–	–	Minutes
	Leadership and work environment		T1, T2	T2	T0a, T2	–	–	–	Questionnaires (PES Nursing Work Index, NoMAD), minutes
	Financial and staff resources		T1, T2	T0a, T2	T0a, T2	–	T0a, T2	–	CRF, questionnaires, minutes
	Structures of collaboration and medical care	Attitudes, current practice, quality, and satisfaction	T1, T2	T0a, T2	T0a, T2	–	T0a, T2	–	Questionnaires (InDemA adapted)
	GP office and NH characteristics		–	–	T0a, T2	–	T0a, T2	–	CRF, questionnaires (Koverdem), minutes
Micro: staff level	Sociodemographic characteristics of all persons involved in implementation		T0b	T0a, (T0b), T2	–	–	T0a, (T0b), T2	T0a, (T0b)	Questionnaires, minutes
	Expected effects of high quality RN-GP collaboration		–	T0a, T2	T0a, T2	–	T0a, T2	–	CRF, questionnaires (Koverdem)
	Competences of RNs, GPs, and IPAV		T2	T0a, T2	T0a, T2	–	T0a, T2	–	CRF, questionnaires (Koverdem), minutes
Micro: NHR level	Utilization of GP office (formal characteristics)		–	–	–	T0a, T2	–	–	Questionnaires (ZAP)
	Family involvement in medical care		–	–	–	T0a, T2	–	–	Questionnaires (Koverdem)
Multiple levels	Unmet implementation support needs (IPAV)		T1, T2	–	–	–	–	–	Questionnaires
	Other factors (open-ended questions)		T0b, T1, T2	T0a, T2	T0a, T2	–	T0a, T2	T1, T2	Questionnaires, minutes

Instruments: Zufriedenheit in der Arztpraxis (“Satisfaction in GPs office”, ZAP) [25]. Survey instruments of the research project “Optimization of the cooperation between general practitioners and home care services” Koverdem [26]. Measures of the European Project on Patient Evaluation of General Practice Care (EUROPEP) [27]. Measures of interprofessional collaboration designed for the pre-post evaluation study “Interdisciplinary Implementation of Quality Instruments for the Care of residents with Dementia in Nursing Homes” (InDemA) [28]. Normalization Measure Development QUES (NoMAD) [29, 30]. Partnership Self-Assessment Tool (PSAT) [31]. Jefferson Scale of Attitudes toward Physician–Nurse Collaboration [32]. Collaborative Practice Assessment Tool (CPAT) [33]. Practice Environment Scale of the Nursing Work Index (PES-NWI) [34, 35]

*Abbreviations*: *CRF* case report form, *GPs* general practitioners, *IPAVs interprof* ACT agents, *NHD* nursing home directors, *NHRs* nursing home residents, *T0a* before randomization, *T0b* after randomization, *(T0b)* as participants and moderator of kick-off meeting, *T1* follow-up after 6 months, *T2* follow-up after 12 months, *RNs* registered nurses, *STM* study team member

^a^Single items or subscales of reported tools are used for the outcome domains in question. In addition, self-developed items are used for the measurement of all outcome domains

The major outcome domain “implementation strategies and activities” includes various dimensions (e.g., quality and satisfaction) of the implementation of the kick-off meetings and the IPAV training and the IPAV supervision. These dimensions will be assessed from the perspectives of the groups targeted by these implementation strategies (e.g., participants of the kick-off meetings, IPAVs) and those responsible for the application of these implementation strategies (e.g., study team members). Data will be gathered by means of semistructured minutes and questionnaires consisting of self-developed items. In addition, information will be collected on the activities undertaken by the IPAVs and the nursing staff to implement the *interprof* ACT interventions. This information will be collected from the IPAVs, RNs, and the study team members by means of standardized questionnaires consisting of self-developed items and items of the Normalization Measure Development (NoMAD) questionnaire [29, 30]. This instrument measures the core constructs of the Normalization Process Theory (NPT), i.e., coherence, cognitive participation, collective action, and reflexive monitoring, which describe and explain critical mechanisms of successful implementation of new complex interventions into the routines of healthcare practice [36, 37]. These constructs emphasize the continuous activities undertaken by involved persons both individually and collectively to make the new intervention meaningful, usable, and useful for their practice, to adapt novel and existing practices to each other and to evaluate and reflect on noticeable effects [36]. The convergent and structural validity and the reliability of the NoMAD questionnaire have been confirmed in several surveys among health professionals [29, 30, 38]. For this process evaluation, items reflecting all four NPT core constructs were chosen from the German version of the NoMAD questionnaire [39] and adapted to the terminology of the *interprof* ACT intervention package (Additional file 2).

The major outcome domain “implementation of the *interprof* ACT intervention package” contains several domains and subdomains to measure the success of the implementation activities in terms of the following dimensions: dose and reach of the intervention package as a whole and of the individual *interprof* ACT interventions, adaptations made to the single interventions, and attitudes and subjectively experienced quality with regard to these interventions. Adaptations made to the *interprof* ACT interventions will be recorded by study team members by completing semistructured minutes of the kick-off meetings and the supervision encounters as well as by IPAVs (questionnaires at T1 and T2). Dose and reach are directly measured by repeated 10-step global judgments of the IPAVs on the degree of implementation (1 = not implemented, 10 = fully implemented). The judgments of IPAVs, RNs, nursing home directors, GPs, and NHRs on attitudes, current practices and/or subjectively experienced quality of/satisfaction regarding *interprof* ACT interventions will provide an indication of the fidelity and quality of the implementation. Respective judgments will be collected at T0 and T2 by means of standardized questionnaires comprising self-developed items and items of existing measurement instruments with established psychometric performance [26, 28–31] (Table 2 and Additional file 2).

Two other major domains, “key elements of interprofessional collaboration” and “further domains related to interprofessional collaboration and medical care,” address the key elements of interprofessional collaboration (e.g., interprofessional communication, involvement of NHRs, contribution of involved professions), additional aspects of collaboration and experiences of quality of care assumed to be influenced by the *interprof* ACT intervention package (Fig. 3). These outcomes will be assessed in terms of attitudes, current practice, and subjectively experienced quality or satisfaction from various perspectives (IPAVs, RNs, nursing home directors, GPs, and NHRs) at different measurement points (T0, T2). Standardized questionnaires consisting of self-developed items and items of established measurement tools [25–28, 31–33] will be used (Table 2 and Additional file 2).

The contextual factors (major outcome domain (5)) comprise domains and subdomains that relate to attributes of the health system, the involved organizations, the *interprof* ACT intervention package, the staff, and the NHRs, which are assumed to have moderating effects on implementation success, interprofessional collaboration, and the quality of medical care for NHRs (Fig. 3). Following the underlying evidence [15], they are classified as factors at the macro-, meso-, and microlevels. Data on these domains and subdomains will be collected from each target group that contains or is affected by the contextual factors of interest. To gather these data, standardized questionnaires, case report forms, and semistructured minutes of supervision encounters will be used. Whenever justified and possible, items of validated measurement tools [26, 29, 30, 34, 35] will be integrated into these data collection instruments, complemented by self-reported items (Table 2 and Additional file 2).

The questionnaires and minute templates were designed based on existing measurement tools used in previous process evaluation studies on complex interventions to improve the quality of care in long-term institutions [8, 40]. The questionnaires for the RNs and GPs consist of two parts: one general part (part A) containing items to be answered independently of collaboration with a certain GP (questionnaires for the RNs) or a certain nursing home (questionnaires for the GPs) and a specific part (part B) that contains items specifically referring to collaboration with a certain GP’s office (questionnaires for the RNs) or a certain nursing home (questionnaires for the GPs). The items of the general part A cover all major outcome domains but do not include items on current practice and the subjectively experienced quality of/satisfaction with interprofessional collaboration and medical care within the major outcome domains “implementation of *interprof* ACT intervention package” and “key elements of interprofessional collaboration.” Additionally, part A does not include items referring to contextual factors that are specific to one collaborating GP office or nursing home (e.g., staff competencies in this institution). Respective outcomes are assessed specifically by part B of the questionnaires for GPs and RNs. Here, GPs are asked to complete a part B questionnaire specifically for each nursing home in which they provide medical care for NHRs participating in this trial. This means that the questionnaires for the GPs include as many parts B as the number of participating nursing homes with which they are currently collaborating. In contrast, the questionnaires for the RNs include specific part B questionnaires only for a 30% random sample of the GPs’ offices currently responsible for medical care for participating NHRs in their nursing home. This approach is used to avoid a large number of part B questionnaires being completed by RNs in nursing homes collaborating with more than six GPs’ offices [8].

Questionnaires for the RNs and GPs were piloted before the T0 measurements by means of cognitive interviews with nursing staff (*n* = 8) of 3 nursing homes and 10 GPs not involved in the main study. Within this pilot study, the completeness and comprehensibility of the questionnaires were assessed, and the relevance of the items of the major outcome domain “key elements of interprofessional collaboration” was determined by means of the content validity index. The pilot study revealed only minor need for revision of the questionnaires. Based on these findings, single items were deleted, added, or rewritten.

All questionnaires are paper-based. The questionnaires for the RNs, IPAVs, and GPs will be completed by the target persons. Participating GPs will receive postal questionnaires together with prepaid opaque return envelopes. Questionnaires for RNs and IPAVs will be directly handed to them by study team members during scheduled follow-up visits. Anonymously completed questionnaires will be collected by the study team members (IPAV questionnaires) or NHD (RN questionnaires). Questionnaires for NHRs will be part of structured oral interviews conducted by study team members for the collection of patient-reported data for the main trial at follow-up visits T0 and T2 [12].

#### Qualitative process evaluation

Data collection for the qualitative part comprises non-participating observations and semistructured interviews. Table 3 presents an overview of the themes considered in interviews and observations.

**Table 3:** Overview of themes considered in interviews and observations of the qualitative process evaluation

Data collection method	Themes
Semistructured interviews	• Process sequences of care, interprofessional collaboration, and hospital admission • Everyday work context of GPs and RNs • Perception of work processes between GPs and RNs • Barriers and facilitators for process performance • Evaluation of process changes through *interprof* ACT intervention (only in IG) • Barriers and facilitators for implementation of *interprof* ACT measures (only in IG) • Feasibility of *interprof* ACT measures (only in IG)
Non-participating observations	• Communication and interaction between participants during kick-off meeting (only in IG) • Tailoring of *interprof* ACT measures during kick-off meeting (only in IG) • Process sequences of care, interprofessional collaboration, and hospital admission • Communication between GPs and RNs • Barriers and facilitators for implementation of *interprof* ACT measures (only in IG) • Evaluation of process changes through *interprof* ACT measures (only in IG) • Feasibility of *interprof* ACT measures (only in IG)

*Abbreviations*: *GPs* general practitioners, *IG* intervention group, *NHRs* nursing home residents

For the observations, one or two researchers will visit each of the eight participating nursing homes at T0, T1, and T2 to observe GP-RN interaction and communication as well as the implementation of the *interprof* ACT intervention and associated work process changes in the intervention group. Short conversations with GPs and RNs will be held before or after the observations if possible. The participation of NHRs is not intended but may be considered in relevant situations of communication between GPs, RNs, and NHRs. In addition to these observations, one researcher will attend the kick-off meetings to observe the interaction and communication between GPs and RNs when choosing and tailoring *interprof* ACT intervention components. Observations will be carried out with semistructured observational guidelines [41]. The observers will appear explicitly as scientists, and there will be no active participation of observers in the situation [42]. Observers will take notes while observing. The notes will be pseudonymized and then stored in a word processor (e.g., Microsoft Word).

In addition to the non-participating observations, interviews will be conducted with GPs and RNs. These interviews complement the observation data for the description of the care process and the evaluation of process changes through the *interprof* ACT intervention package in the intervention group. Furthermore, they serve to clarify observations and assess respondents’ perceptions of the processes. Semistructured interview guidance will be developed by the researcher team consisting of open questions that allow an unbiased and flexible approach. Interviews will be conducted by one interviewer with one respondent at a time. Interviews will take place in the nursing homes or at any other place chosen by the interview partners. Interviews will be audio-recorded and subsequently transcribed and pseudonymized. If the interview partners prefer that the interview will not be recorded, the interviewer will take notes. During the data collection process, the research team will regularly discuss the initial findings and adjust the interview guidelines to account for emerging themes.

For observations, an overall time frame of 1 to 3 days at 1 to 3 h a day per nursing home per measurement point is expected. Approximately four interviews involving one or two GPs and two RNs are planned per nursing home per measuring point.

### Data management

Study data in the process evaluation will be recorded both pseudonymized (Nursing or facility directors, NHRs) and anonymized (RNs, GPs, IPAVS, and study team members). For pseudonymized data, the personal data of the study participants will be kept separately from the study data. A retrospective correlation to a person is only possible with the help of a “key” which is maintained in the study center. The data will be entered into the electronic database according to the four-eye principle. The paper-based data is stored in the participating study centers and electronically in the database on a server. To ensure data quality, a plausibility check of the data will be carried out by an independent monitor. All original data will be stored for 10 years and destroyed afterwards. Generally, all data will be handled according to current data protection law.

### Data analysis

Data will be analyzed in a stepwise manner. First, quantitative and qualitative data will be analyzed independently from each other, followed by iterative triangulation. All analyses will be explorative, framed by the underlying logical model (Fig. 3). Statistical analyses will be performed using IBM SPSS Statistics (version 20) and Microsoft Excel. Software packages of MAXQDA (VERBI GmbH) and SAP Signavio will be used to facilitate the management, analysis, and visualization of qualitative data.

#### Quantitative data

To answer the three research questions of the quantitative process evaluation, data on outcome domains for each measurement point will be analyzed by means of descriptive statistics: frequencies (proportions) in the case of categorical variables and measures of location (mean and median) and dispersion (standard deviation (SD) and interquartile range (IQR)) in the case of ordinal or continuous variables. If psychometrically justified, items referring to the same outcome subdomain or dimension will be summarized into one sum score. Only items using the same answer scale will be considered for this aggregation, and the structural validity and reliability will be tested by means of exploratory factor analyses (principal component analysis) and Cronbach’s alpha. Newly created sum-scores will be maintained if each item loads with an eigenvalue of ≥0.25 on the one factor assumed a priori and/or the Cronbach’s alpha is above 0.4. These thresholds are specifically defined for the purpose of this study, taking statistical conventions for estimation and interpretation of these measures into account [43] and assuming that in many cases, not more than five items will be eligible for combination into one sum score.

Initially, all quantitative data will be analyzed and aggregated at the cluster (i.e., nursing home) level. For this aggregation, a two-step approach will be used. First, the direction and size of changes over time will be descriptively estimated for single items or subscales. For the assessment of pre-post changes (T0 versus T2), the difference between the two mean or median values will be estimated. Following the effect size Cohen’s *d* [44], a mean difference with a size of ≥20% of the standard deviation across study groups will be interpreted as a relevant change or difference. For median values, a median difference of 0.5 in case of ≤5-step scales or 1.0 in case of >5-step scales will be regarded as relevant. Change estimates will be classified as follows: relevant changes in the favorable direction reflect positive changes, relevant changes in the nonfavorable direction reflect negative changes, and nonrelevant changes are interpreted as indifferent effects (no changes). Some items will be assessed only at the T2 follow-up visit to directly measure the subjectively perceived degree of change in certain collaboration or care procedures. For these items, the size of the change will be classified based on the distance between the estimated mean value and the most neutral value of the scale. If the mean value is higher (positive values) or lower (negative values), the distance between 20% of the pooled standard deviation and the most scale-neutral value, a relevant change will be assumed, either positive or negative, depending on the direction. In other cases, the mean values will be regarded as being indifferent.

Second, the change estimates across items and subscales will be aggregated by means of vote counting, i.e., the number of positive and negative changes and indifferent findings will be counted and visually depicted in tables for single-outcome subdomains or dimensions. Vote counting findings from each perspective (NHRs, NHDs, RNs, IPAVs, GPs) will be summarized per nursing home in tables (intervention group only), and nursing homes will then be ranked with regard to their degree of implementation achieved for each *interprof* ACT component. Based on this ranking, nursing homes will be inspected for differences in contextual factors. These findings will then inform the triangulation of qualitative and quantitative data and the subsequent creation of a preliminary model to answer the second research question, i.e., the impact of the degree of implementation on the quality of interprofessional collaboration, taking moderating effects of potentially relevant contextual factors into account.

Furthermore, for the outcome domains “implementation of the *interprof* ACT intervention package,” “key elements of interprofessional collaboration,” and “further domains related to interprofessional collaboration and medical care,” the vote counting findings per nursing home will be visually compared between the intervention and control groups to explore between-group differences in the changes in attitudes and current practice and the quality of RN-GP collaboration.

#### Qualitative data

Qualitative data analyses will serve to explore processes and identify intervention-induced process changes in the intervention group based on qualitative content analysis procedures [42]. First, individual cases will be analyzed to describe the process patterns of time-ordered events (including involved actors and contextual factors) for each nursing home at different measurement points using process modeling techniques. Cases will then be compared to note similarities and differences across cases and to identify intervention-induced changes. Effective changes will be described and defined in a standardized form. To ensure reliable analysis of qualitative data, it will be conducted by two coders (i.e., two researchers) who independently assign codes, which will then be compared and discussed by the research team. In the course of this iterative process, the aim will be to identify relevant process changes and to reveal specific process patterns across cases that enable the development of general process descriptions by using process maps.

#### Triangulation

In addition to the separate analyses of quantitative and qualitative data, synthesis and joint analysis of quantitative and qualitative findings are planned to enhance the quality of the process evaluation and to evaluate and adapt the underlying logic model in accordance with the empirical findings. Concretely, triangulation will allow us to explore the impact of the degree and fidelity of the implementation of the *interprof* ACT intervention package on the quality of interprofessional collaboration, the interaction between contextual factors and the success of implementation, and the quality of interprofessional collaboration.

For this purpose, quantitative and qualitative findings will be integrated at different timepoints. First, after collecting initial T0 data sets, preliminary qualitative insights regarding the determinants of interprofessional collaboration and contextual factors will be exchanged and discussed against preliminary experiences derived from the supervision of IPAVs to allow for the adjustment of subsequent T1 and T2 questionnaires for emerging themes.

Second, after completion of the T2 data collection, the findings of the separate quantitative and qualitative analyses will be systematically cross-mapped in “joint displays” to compare and contrast insights. This procedure allows us to consider detailed variations within and across clusters (i.e., nursing homes) regarding implementation success, the quality of RN-GP collaboration, and potentially relevant contextual factors. In particular, this step enables a data-driven selection of variables for a model that, based on the a priori logic model, depicts the associations between these variables. To assess the direction and size of the impact of the implementation success on important variables of RN-GP collaboration, this model will be subject to generalized linear regression analyses. These analyses will both adjust for cluster-related effects and account for moderating effects of potentially relevant contextual factors. The exact regression model will be chosen based on the actual size and characteristics of available data. By ensuring that quantitative and qualitative analyses build upon each other [17], this triangulation facilitates the creation of a theoretically and empirically informed model for the final statistical exploration of core associations of assumed causal pathways of the *interprof* ACT intervention package.

## Discussion

This process evaluation integrates a variety of qualitative and quantitative data sources that span the perspectives of all stakeholders involved in the *interprof* ACT trial. It follows an evidence-based, theoretically underpinned logic model that illustrates the assumed causal pathway of the *interprof* ACT intervention package, including critical intermediate outcomes (mediators) and potentially relevant contextual factors (moderators). The basic assumption of the *interprof* ACT trial is that the consequential implementation of the *interprof* ACT intervention components will improve key elements of RN-GP collaboration, such as the quality of communication between these two professions (e.g., in the context of GPs’ nursing home visits) or shared goal setting, care planning, and coordination [11], particularly in view of likely or actually occurring deterioration of NHRs’ individual health conditions. It is further assumed that these improvements in RN-GP collaboration will lead to optimized procedures of medical care for NHRs and thus prevent unnecessary utilization of hospital or emergency care services. However, recent trials on the effectiveness of complex interventions pursuing similar goals as the *interprof* ACT trial (i.e., the prevention of unplanned hospital admissions or emergency care utilization through adaptations to interprofessional care pathways for NHRs) have shown mixed results [45, 46]. In secondary analyses of one of these trials, the INTERACT trial, a marked dose–response relation was observed, indicating that nursing homes that achieved higher degrees of implementation of the complex intervention reported lower rates of all-cause hospitalizations compared to nursing homes with poor implementation success [47]. The same analyses also suggest that successful implementation of the INTERACT measures was largely driven by the motivation of nursing homes to implement quality improvement strategies rather than by the implementation strategies applied in this trial.

These findings underscore the limited predictability of the exact causal pathways and contextual factors that determine the downstream effects of complex interventions targeting the dynamic interplay of organizational conditions, professionals’ behavior, and NHRs’ preferences and needs that interact in shaping interprofessional care delivery to NHRs [48]. The findings also highlight the need for a systematic and comprehensive process evaluation alongside the evaluation of the clinical effectiveness of such interventions. Therefore, we expect this mixed methods study to provide valid accounts of the implementation of the *interprof* ACT intervention package, the changes induced in interprofessional collaboration and the influence of contextual factors.

While the *interprof* ACT process evaluation triangulates quantitative and qualitative data from all parties involved in the delivery and reception of the interventions under evaluation, some potential limitations have to be considered. The qualitative part will be conducted only in a voluntary subsample of nursing homes in the intervention and control groups, thus increasing the risk or recruitment bias due to participation of highly motivated facilities. However, since recruitment strategies for the qualitative inquiries will strive for a heterogeneous sample in each study group and the risk of recruitment bias may be equally high across the intervention and the control groups, likely selective participation may affect the credibility of the process evaluation findings only to a minor degree. Furthermore, patient-reported data on the NHRs’ perception of the collaboration between the RNs and the GPs and their satisfaction with the quality of received medical care will be limited to those able to speak for themselves. Proxy assessment of these patient-reported experiences by nursing staff is considered as being not justified, and inclusion of family caregivers were regarded as being not feasible for logistical reasons. However, despite these limitations this process evaluation will cover a broad spectrum of perspectives and reveal expected and unexpected changes in the procedures of interprofessional care delivery. Thus, the findings of this process evaluation will facilitate accurate interpretation of the main trial findings and allow for robust conclusions concerning the further design of routine care services for NHRs. They will also improve our understanding of the mechanisms of interprofessional collaboration and their impact on the quality of care for vulnerable people such as NHRs.

### Trial status

Recruitment and data collection are finished, and data analysis is ongoing.

## Supplementary Information

**Additional file 1. **Description of the intervention following the Template for Intervention Description and Replication (TiDier) criteria [1]; Characteristics of the *interprof* ACT intervention package (description based on the TIDieR checklist).

**Additional file 2. **Overview of standardized instruments used for the quantitative part of the *interprof* ACT process evaluation; Instrument names, measured outcome domains and subdomains, and description of item and scaling formats.

## Data Availability

Data are available upon reasonable request to the publication committee of the *interprof* ACT project.

## Abbreviations

NHR: Nursing home resident
GP: General practitioner
RN: Registered nurse
STM: Study team member
NHD: Nursing home director
c-RCT: Cluster-randomized controlled trial
T0: Baseline data collection
T1: Follow-up after 6 months
T2: Follow-up after 12 months
StaRI: Standards for Reporting Implementation Studies Statement
IPAV: interprof ACT agent
NoMAD: Normalization Measure Development
NPT: Normalization Process Theory
ZAP: Zufriedenheit in der Arztpraxis
Koverdem: Survey instruments of the research project “Optimization of the cooperation between general practitioners and home care services”
EUROPEP: Measures of the European Project on Patient Evaluation of General Practice Care
InDemA: Measures of interprofessional collaboration designed for the pre-post evaluation study “Interdisciplinary Implementation of Quality Instruments for the Care of residents with Dementia in Nursing Homes”
PSAT: Partnership Self-Assessment Tool
CPAT: Collaborative Practice Assessment Tool
PES-NWI: Practice Environment Scale of the Nursing Work Index
SD: Standard deviation
IQR: Interquartile range
TIDieR: Template for Intervention Description and Replication
