# Supplementary material for: Implementation, mechanisms of change and contextual factors of a complex intervention to improve interprofessional collaboration and the quality of medical care for nursing home residents: study protocol of the process evaluation of the interprof ACT intervention package
Source: Trials. 2022 Jul 8;23:561. doi: 10.1186/s13063-022-06476-6 (PMC9270799; doi:10.1186/s13063-022-06476-6)
Supplement: Supplementary file 1 — Additional file 1. Description of the intervention following the Template for Intervention Description and Replication (TiDier) criteria [1]; Characteristics of the interprof ACT intervention package (description based on the TIDieR checklist). [file 13063_2022_6476_MOESM1_ESM.docx]

**Additional file 1: Description of the intervention following the Template for Intervention Description and Replication (TiDier) criteria** [1]

**1. Brief Name**

*interprof* ACT trial: Effects of strategies (*interprof* ACT intervention package) to improve general practitioner-nurse (interprofessional) collaboration and communication in regard to hospital admissions of nursing home residents (NHRs).

**Why**

The implementation of the *interprof* ACT intervention package is thought to improve collaboration and communication between general practitioners (GPs) and registered nurses (RNs) in long-term care facilities and consequently reduce hospital admissions of NHRs. Up to 50% of NHRs experience hospital admission for an average of 12 months in Germany [2-4], of which approximately 30% might be avoidable [5]. One very important element in interventional studies in the nursing home setting for a positive impact on NHRs´ health is the involvement of NHRs´ GPs [6]. In a previous study, the *interprof* ACT intervention package was developed on the basis of interviews, focus groups and an expert workshop with involved person groups (NHRs, nurses, GPs and relatives). A pilot study found the *interprof* ACT intervention package to be feasible [7].

**3./4. What (Materials/Procedures)**

The *interprof* ACT intervention package is considered a complex intervention since it contains multiple components that could be individually combined and adapted to local conditions by participating nursing homes and GPs´ offices [8] (Table S1). All components are to be implemented for twelve months. A description of the components of the *interpro*f ACT intervention package is available as an open-source publication [7].

Several strategies are used to facilitate the implementation of the *interprof* ACT intervention package. The core strategies are as follows:

**Designation and training of *interprof* ACT agents**

In each nursing home, an *interprof* ACT agent and a substitute are designated. The tasks of these agents are to inform the staff about the *interprof* ACT study and intervention package and to initiate, coordinate and monitor local activities for the implementation of the components in the nursing home. Moreover, these agents stay in close contact with the research team, which regularly provides supervision (see strategy (5)). *Interprof* ACT agents are designated by the nursing home management. To be nominated as an *interprof* ACT agent or a substitute, staff members must fulfill each of the following criteria: 1) being qualified as a registered nurse (three-year vocational training in geriatric or general nursing), 2) working mainly day shifts, 3) being present at least 20 hours per week on average, 4) having regular contact with visiting GPs and 5) having good knowledge of the NHRs’ health situation. One *interprof* ACT agent and one substitute are designated per nursing home [8].

*Interprof* ACT agents receive all intervention materials (Table S1) during two individual training sessions delivered by local study team members. The first training (120 min) aims to inform the *interprof* ACT agents about the study, the objectives, components and procedures of the intervention package and their own roles and tasks. In particular, the *interprof* ACT agents are instructed about the purpose, target populations and procedures of the kick-off meeting (see strategy 2) since they will have to organize, moderate and analyze this meeting. The second training (120 min) is conducted within 1–2 weeks after the kick-off meeting and aims to pave the way for the local implementation of the *interprof* ACT intervention components as agreed upon during the kick-off meeting. The subjects of this training are (1) discussion of the kick-off meeting outcomes, (2) identification and reflection of potential local facilitators and barriers to successful implementation, and (3) next steps for local implementation, including activities to be initiated by the *interprof* ACT agent. To strengthen the *interprof* ACT agents’ self-confidence in their change agent competencies, this second training addresses their reflection and communication skills and includes role play to simulate typical challenging situations in the implementation process.

**Kick-off meeting in the nursing home with all involved parties**

Within 4–5 weeks after the randomized allocation, an in-house kick-off meeting is held at each nursing home assigned to the intervention group. At these meetings, the *interprof* ACT intervention package is introduced to and discussed among all involved parties, i.e., the nursing or facility director, registered nurses of participating nursing home units, GPs in charge of included NHRs, NHRs, legal guardians, representatives of the nursing homes´ advisory board, and members of the relatives´ advisory board or up to two interested relatives as representatives. The aim is to agree on the shape of the *interprof* ACT intervention components to be implemented in the nursing home. Based on existing structures and procedures for nurse-GP collaboration and medical care for NHRs in the respective nursing homes, single components of the intervention package can be chosen, omitted or adapted to local needs.

In preparation for the kick-off meeting, all participants receive three of the abovementioned materials: a pro re nata medication form, a meeting form (for meetings to establish common goals) and a standardized fax form. Furthermore, the nurses of participating nursing home units rate the local relevance and feasibility of the single components in advance, and these ratings (yes, no, which) are taken into account during the kick-off meeting discussion. At the kick-off meeting, each component is discussed until all participants agree.

The *interprof* ACT agents organize the kick-off meeting with the support of the nursing and facility director and the study team. GPs are invited by the study team. *Interprof* ACT agents invite all other potential participants.

**Involvement of NHRs´ GPs**

All GPs of the participating NHRs are invited by members of the research team to take part in the kick-off meeting. They receive written information about the components of the *interprof* ACT intervention package and the name of the *interprof* ACT agent. During the kick-off meeting, they co-shape the components together with the other participants.

**Involvement of NHRs**

NHRs and their relatives or legal guardians are informed about the assignment of the respective nursing homes.

**Regular supervision contact of *interprof* ACT agents with research team members**

Members of the local study team will regularly supervise the *interprof* ACT agent via telephone/electronic contact and face-to-face meetings and help her/him reflect on the advancements and barriers occurring in the implementation of agreed-upon *interprof* ACT components. For the first three months after the kick-off meeting, two to four telephone/electronic contacts per month and one to three face-to-face meetings in total are planned, which should be reduced to one to two monthly telephone/electronic contacts and one face-to-face meeting every other month in the remaining study period. The exact time points and intervals of contacts are at the discretion of the designated *interprof* ACT agents and the supervising study assistant. Additionally, *interprof* ACT agents are invited to contact their contact person on the local research team at any time if questions or problems arise. With the help of this supervision, *interprof* ACT agents are expected to identify and implement locally targeted implementation strategies. All supervising study members hold a bachelor’s degree in a health profession at minimum and are familiar with the conditions of long-term nursing care for the elderly in Germany.

Table S2 shows the chronological order of the implementation steps and the materials used in each step. The materials developed for the *interprof* ACT components (Table S1) and their implementation (Table S2) are not available to the public during the study period.

**Table S1**. Components of the *interprof* ACT intervention package

| **Components of intervention package** | **Description of the components** | **Target persons** | **Persons responsible for delivery or application** | **Location** | **Modes of delivery** | **Frequency, dosage and duration** | **Materials used**  **(provided by the research team)** |
| --- | --- | --- | --- | --- | --- | --- | --- |
| **Use of name badges by GPs and nurses** | During GPs´ visits in the nursing home, nurses and GPs wear name badges. | GPs, Nursing staff | GPs, Nursing or facility director,  Nurses | Nursing home | Not applicable | During GPs´ visits | **Name badges** are provided by the nursing homes. |
| **Fixed contact person** | Nursing homes appoint one registered nurse per day shift per unit as primary GP contact.  GPs appoint one member of their office staff as permanent contact person. | GPs and their offices, RNs | GPs and their offices, Nursing facility director, RNs | GPs´ office, Nursing home | Not applicable | Not applicable | **Door notice**  Indicates name of the nurse currently acting as primary GP contact  **Abstract of GP contacts for nurses**  table includes name of GP, opening hours of GPs´ office, fax number, telephone number and if applicable name of appointed contact person in GPs´ office (also related to component “mandatory availability”). |
| **Mandatory availability** | **Via telephone**  Portable telephones are used in nursing homes, answering machines are listened to regularly.  **Via fax**  Fax should only be sent during office hours of GPs. Fax machines are accessible during the entire day by nurses. Standardized fax forms are used. | GPs, Medical assistants (GPs´ office staff),  RNs | GPs, Medical assistants (GPs´ office staff),  RNs | Nursing home | Not applicable | On demand | **Standardized fax form** contains several rubrics, for example, reason for the nurse’s request to the GP including clinical core details of the triggering incident, answer from GP, implementation by nurse, detailed information on contact person, urgency of an answer, affirmation of reading. |
| **Standardized procedures for GPs´ home visits** | **Scheduling of the visit:**  GP visits will either be scheduled weekly or be announced two days in advance and arranged within a time slot of about two hours.  If relatives attend, the appointed contact person (registered nurse) informs them about the schedule of the visit.  **Structure of the visit:**  The appointed contact person of the unit collects and prioritizes the current clinical concerns regarding the NHRs of interest in advance.  On arrival, GP and the appointed nurse discuss these concerns.  The actual home visit might be accompanied by the appointed contact person (registered nurse) or not.  At the end of the visit in the nursing home, the GP and the appointed nurse discuss further procedures or the GP provides clear instructions in written form if no nurse accompanied the GP. Instructions are documented in NHRs´ files. | GPs, RNs, NHRs,  *if desired* relatives or legal guardian | GPs, RNs,  (Nursing or facility director who may have to adjust the staff roster to GPs´ visiting times) | GP´s office, Nursing home | Not applicable | Not applicable | No materials were provided for this component. |
| **Assignment of pro re nata medication** | Specification of pro re nata medication for each NHR by the respective GP for 12 months. | GPs, RNs, NHRs | GPs, RNs | Nursing home | Not applicable | Not applicable | **Pro re nata medication form**  contains details on medical indication, dosage and maximum daily dose of pro re nata medication for individual NHR. |
| **Meetings to establish common goals** | Overarching and longer-term resident-specific goals for medical care defined and documented by all involved parties (see target persons).  The goals and wishes of the NHRs are in focus. | GPs, RNs, NHRs, *if desired* relatives or legal guardian | GPs, RNs | Nursing home | Face-to-face meetings | Quarterly | **Preparation card for nurses and GPs**  gives information on the process and potential fields to consider  **Preparation form for NHRs** explains the meeting and invites NHRs to present important issues from their own perspective  **Meeting form** for the documentation of core meeting data, such as date of meeting, involved persons, approved goals, further procedure and tasks of involved persons and date of next meeting |

RNs = Registered nurses. NHRs = Nursing home residents. GPs = General practitioners.

**Table S2**. Strategies designed for the implementation of the *interprof* ACT intervention package

| **Time point** | **Description of the activity** | **Target persons** | **Persons responsible for delivery or application** | **Location** | **Modes of delivery** | **Frequency, dosage and duration** | **Materials used**  **(provided by the research team)** |
| --- | --- | --- | --- | --- | --- | --- | --- |
| At the beginning of the study | **Invitation to take part in the study**  to all nursing homes and GPs of participating NHRs | Nursing or facility director, GPs | Study team members | Not applicable | Written letter and contact by telephone | Not applicable | **Brief written overview** of study design (in intervention and control group) and the six components of the *interprof* ACT intervention package (only in intervention group) |
| Shortly after randomization | **Designation of *interprof* ACT agent and one substitute**  **First training of *interpro*f ACT agent**  During the training, the *interprof* ACT agents receive handbook and handouts about the *interprof* ACT intervention package.  **Team meeting in the nursing home**  The *interprof* ACT agent and the *interprof* ACT intervention package are introduced to the nursing team of the involved nursing home unit. The nurses state any concerns they have about the interventions. | RNs  *interprof* ACT agents  *interprof* ACT agents, nursing or facility director | Nursing or facility director, RNs  Study team members  *interprof* ACT agents, nursing or facility director | Nursing home  Nursing home  Nursing home | Not applicable  Face-to-face session  Group meeting | One-time, but to be repeated if *interprof* ACT agents are changed  One time, 1-2 weeks after randomized allocation to intervention group, duration 120 minutes.  Individually depending on nursing home | **Handbook** provides information about study design and goals, detailed information about the six components, potential material to be used as well as information and checklists for the preparation, organization and facilitation of the kick-off meeting  **Handouts of presentation** summarize the content of the handbook.  **Poster of *interprof* ACT intervention package**  lists components in a table; nurses are invited to assign their rating for each intervention component in written form on the poster (yes, no, which). |
| Kick-off meeting | **Discussion of the *interprof* ACT intervention package** until participating stakeholders (representatives of GPs, nursing staff, NHRs and relatives, nursing home management) find consensus about the components (types of components, adaptations of the components) to implement in the nursing home.  Prior to the meeting, all participants receive written information about the six components of the *interprof* ACT intervention package. | Nursing or facility director, RNs (maximum 2 of each involved nursing home unit), *interprof* ACT agent and substitute, GPs (and *if desired* medical assistants of GPs´ office,  NHRs (maximum 2), relatives (maximum 2) | *interprof* ACT agents, nursing or facility director, study team members | Nursing home | Group meeting | One time, 3 weeks after first training session, duration 120 minutes | **Overview of the six components of the *interprof* ACT intervention package, agenda of the meeting** |
| Shortly after  kick-off meeting | **Preparation of written summary of selected and adapted *interprof* ACT intervention components** for participants of the kick-off meeting and GPs of participating NHRs with interest in study  **Second training of *interprof* ACT agent,**  interprof ACT agents’ presentation during the training | Not applicable  *interprof* ACT agents | Study team members  Study team members | Not applicable  Nursing home | Not applicable  Face-to-face session | Not applicable  One time, 1–2 weeks after kick-off meeting, duration 120 minutes. | **Overview of selected components of the *interprof* ACT intervention package and agreed-upon adaptations**  **Amendment to the handbook:**  Detailed description of the agreed-upon amendments to the intervention components |
| During follow-up supervision | **Regular contacts between *interprof* ACT agents and a member of the research team** to supervise the implementation process and the maintenance | *interprof* ACT agents | Study team members | Nursing home (if face-to-face meeting) | Via telephone or e-mail, and  face-to-face meetings | **First three months after randomization:** monthly 2-4 telephone/electronic contacts and 1-3 face-to-face meetings  **Remaining study period:** monthly 1-2 telephone/electronic contacts and 1 face-to-face meeting | **Checklist for adapted components**  The *interprof* ACT agents are provided with a checklist based on the components selected and adapted in the kick-off meeting. The checklist can be used to note whether and to what extent the tasks have already been implemented.  **Reflection sheet in preparation for regular telephone/electronic contacts** contains the questions that are asked by the study team members during the contacts. |

RNs = Registered nurses. NHRs = Nursing home residents. GPs = General practitioners.

1. **Who provides**

The inhouse implementation and maintenance of the *interprof* ACT intervention package is provided by the *interprof* ACT agents and their substitutes. They receive no incentives or expenditure allowance for their work in the study.

In addition to the *interprof* ACT agents, other persons, such as nursing home managers, nursing staff, NHRs and their relatives, GPs and study team members, are involved in the implementation of the *interprof* ACT intervention package (Table S2). GPs and nursing homes receive an expenditure allowance of EUR 150 per included NHR when allocated to the intervention group and EUR 50 per NHR when allocated to the control group. Participating NHRs receive a small thank you gift.

1. **How**

The modes of implementation of the *interprof* ACT intervention package vary across the implementation strategies and intervention components. For example, the implementation strategies include on-site group meetings (kick-off meetings) targeting different stakeholders, on-site trainings of the *interprof A*CT agents, regular remote contacts via telephone or e-mail and on-site face-to-face meetings between the study team and single *interprof A*CT agents (Table S2). Some of the intervention components require adaptations of existing documentation forms, while others involve direct communication between nurses, GPs and/or NHRs (Table S1).

**7. Where**

NHRs are recruited in 34 nursing homes in three locations in Germany (Göttingen, Hamburg, Lübeck). All GPs of participating NHRs in the control and intervention groups are invited to take part in the study. The intervention is implemented in half of the nursing homes (n=17) randomly assigned to the intervention group and associated GPs´ offices. The majority of the intervention components must be implemented in the nursing homes since these are the places where the NHRs live and are cared for by the nursing home staff and visiting GPs (Tables S1 and S2). In the GPs´ offices, standardized fax forms may have to be newly implemented or adapted, and fixed contact persons must be nominated as part of the implementation of the intervention (Table S1).

**8. When and how much**

The adapted *interprof* ACT intervention package should be implemented after the kick-off meeting, and this implementation should be maintained until the 12-month follow-up after randomized allocation. The frequency and length of single intervention components, such as meetings to establish common goals, must be adjusted to the individual needs of NHRs or local conditions (Table S1).

**9. Tailoring**

The *interprof* ACT intervention package can be adjusted to the local requirements and needs of all parties involved in the GP-nurse collaboration and medical care for the NHRs in the respective nursing homes. These adaptations may include changes to the shape, frequency, length or intensity of intervention components or individually tailored materials. Additionally, all involved parties may agree to not implement one component, either because a similar procedure or structure is already in place or because the intervention is viewed as not justified or feasible. Local changes to the intervention package should be discussed and agreed upon by all parties involved in the kick-off meeting immediately after randomized allocation (see more detailed information in sections 3/4).

**10. Modifications**

The *interprof* ACT intervention package or the implementation strategies might be modified if necessary. Any modification will be noted by the process evaluation.

**11. How well (planned)**

Several strategies are used to maintain fidelity in this trial. First, *interprof* ACT agents are trained twice by members of the local research team. This training also includes the handling of problems that may occur during the implementation, especially with regard to noncompliance by nursing home staff. Second, the implementation process and maintenance are supervised regularly by one (if possible, always the same) member of the local research team (see sections 3 and 4 and Table S2). For this purpose, individual checklists are developed as an aid for the *interprof* ACT agent to assess the degree of implementation and barriers that occur each month. They share these assessment results with the supervising member of the study team and discuss strategies to overcome the identified barriers. Aside from this supervision, study team members are not actively involved in the implementation of agreed-upon intervention components.

**12. How well (actual)**

Assessment of the dose, reach and fidelity of implementation and of any changes made to the intervention components is part of the process evaluation. The results of this evaluation will be reported when data collection and analyses for this evaluation are completed but not later than 18 months after the last patient out.

**References**

1. Hoffmann TC, Glasziou PP, Boutron I, Milne R, Perera R, Moher D, et al. Better reporting of interventions: template for intervention description and replication (TIDieR) checklist and guide. BMJ. 2014;348:g1687.

2. Gerster B, Günster C, Bartholomeyczik S. Versorgungs-Report 2012: schwerpunkt: gesundheit im alter. Stuttgart: Schattauer; 2012.

3. Schneekloth U, von Törne I. MuGIV: möglichkeiten und grenzen selbständiger lebensführung (MuG IV) integrierter abschlussbericht, bundesministerium für familie, senioren, frauen und jugend. 2008. https://www.bmfsfj.de/blob/78928/9465bec83edaf4027f25bb5433ea702e/abschlussbericht-mug4-data.pdf. Accessed 30 Mar 2021.

4. Hoffmann F, Schmiemann G. Influence of age and sex on hospitalization of nursing home residents: a cross-sectional study from Germany. BMC Health Serv Res. 2017;17:55.

5. Leutgeb R, Berger SJ, Szecsenyi J, Laux G. Potentially avoidable hospitalisations of German nursing home patients? A cross-sectional study on utilisation patterns and potential consequences for healthcare. BMJ Open. 2019;9:e025269.

6. Nazir A, Unroe K, Tegeler M, Khan B, Azar J, Boustani M. Systematic review of interdisciplinary interventions in nursing homes. J Am Med Dir Assoc. 2013;14:471-8.

7. Müller CA, Fleischmann N, Cavazzini C, Heim S, Seide S, Geister C, et al. Interprofessional collaboration in nursing homes (interprof): development and piloting of measures to improve interprofessional collaboration and communication: a qualitative multicentre study. BMC Fam Pract. 2018;19:14.

8. Müller C, Hesjedal-Streller B, Fleischmann N, Tetzlaff B, Mallon T, Scherer M, et al. Effects of strategies to improve general practitioner-nurse collaboration and communication in regard to hospital admissions of nursing home residents (interprof ACT): study protocol for a cluster randomised controlled trial. Trials. 2020;21:913.
