# Supplementary material for: Implementation, mechanisms of change and contextual factors of a complex intervention to improve interprofessional collaboration and the quality of medical care for nursing home residents: study protocol of the process evaluation of the interprof ACT intervention package
Source: Trials. 2022 Jul 8;23:561. doi: 10.1186/s13063-022-06476-6 (PMC9270799; doi:10.1186/s13063-022-06476-6)
Supplement: Supplementary file 2 — Additional file 2. Overview of standardized instruments used for the quantitative part of the interprof ACT process evaluation; Instrument names, measured outcome domains and subdomains, and description of item and scaling formats. [file 13063_2022_6476_MOESM2_ESM.docx]

**Additional file 2: Overview of used standardized instruments used for the quantitative part of the process evaluation**

| **Instrument (References)** | **Language version used** (Reference for translated versions) | **Outcome domains and subdomains of the process evaluation** | **Items and scaling formats** |
| --- | --- | --- | --- |
| *Collaborative Practice Assessment Tool (CPAT)* [1] | English* | Key elements of interprofessional collaboration: Interprofessional communication (quality and satisfaction) | Statements (e.g. “Our team meetings provide an open, comfortable, safe place to discuss concerns.”) to be rated by means of a 4-step rating scale ranging from 1= “completely agree“ to 4=“do not agree” |
|  |  | Key elements of interprofessional collaboration: Contribution of involved professions (current practice, quality and satisfaction) | Statements (e.g. “Relevant information relating to changes in patient/client status or care plan is reported to the appropriate team member in a timely manner.”) to be rated by means of a 5-step rating scale ranging from 1=”very often” to 5=”never” |
| Measures of the *European Project on Patient Evaluation of General Practice Care (EUROPEP)* [2] | German | Further domains related to interprofessional collaboration and medical care: General (medical) care for NHR (quality and satisfaction) | Questions (e.g. “If you think of the medical care you receive here in the nursing home: How often do you have the impression that you get sufficient and timely help when you experience acute health problems?”) to be answered by means of 4-step rating scale ranging from 1=“always“ to 4=“never“ |
| Measures of interprofessional collaboration designed for the pre-post evaluation study *“Interdisciplinary Implementation of Quality Instruments for the Care of residents with Dementia in Nursing Homes” (InDemA)* [3] | German | *interprof* ACT intervention package: Mandatory availability rules (current practice) | Questions (e.g. „How often you do not reach the registered nurse in charge of the patient at your first call?”) To be answered by means of a 5-step rating scale ranging from 1=”very often” to 5=”never” |
|  |  | *interprof* ACT intervention package: Designated contact persons (current practice) | Questions (e.g. „How often a designated contact person has been nominated for you in the nursing home unit of interest?” to be answered by means of a 5-step rating scale ranging from 1=”very often” to 5=”never” |
|  |  | *interprof* ACT intervention package: Standardized home visits (current practice) | Questions (e.g. „How often fix appointments for the nursing home visits are being agreed on by you and the nursing staff of the nursing home unit in advance of the visit?” to be answered by means of a 5-step rating scale ranging from 1=”very often” to 5=”never” |
|  |  | *interprof* ACT intervention package: Shared goal setting (current practice) | Questions (e.g. “How often you are invited to case conferences in nursing homes to discuss the care for nursing home residents together with all persons involved in the care for these residents?“) to be answered by means of a 5-step rating scale ranging from 1=”very often” to 5=”never” |
|  |  | Key elements of interprofessional collaboration: Contribution of involved professions (current practice) | Questions (e.g. “How often do nurses adhere to shared care decisions?“) to be answered by means of a 5-step rating scale ranging from 1=”very often” to 5=”never” |
|  |  | Key elements of interprofessional collaboration: Coordination of care decision and care planning (attitudes, current practice) | Questions (e.g. „ How often do you make shared arrangements for the care for nursing home residents together with the responsible nursing staff?” to be answered by means of a 5-step rating scale ranging from 1=”very often” to 5=”never” |
|  |  | Further domains related to interprofessional collaboration and medical care: General interprofessional collaboration (quality and satisfaction) | Questions (e.g. “How satisfied are you with the collaboration with the nurses in general?”) to be answered by means of a 5-step rating scale ranging from 1=“absolutely“ to 5=“absolutely not“ |
|  |  | Context factors: Meso – organizational level: Structures of collaboration and medical care (attitudes, current practice) | Questions (e.g. “How often is there sufficient time in the nursing home units to discuss current medical care issues with the nurses?“) to be answered by means of a 5-step rating scale ranging from 1=”very often” to 5=”never” |
| *Jefferson Scale of Attitudes toward Physician-Nurse Collaboration* [4-6] | German [7] | Key elements of interprofessional collaboration: Contribution of involved professions (attitudes) | Statements (e.g. ”There are many overlapping areas of responsibility between physicians and nurses.”) to be rated by means of a 4-step rating scale ranging from 1= “fully agree” to 4=”do not agree” |
| Survey instruments of the research project *“Optimization of the cooperation between general practitioners and home care services” (KOVERDEM)* [8] | German | *interprof* ACT intervention package: Mandatory availability rules (current practice) | Questions (e.g. “In urgent cases: How often does it happen that you reach the general practitioner during out of office hours?“) to be answered by a 5-step rating scale ranging from 1=”very often” to 5=”never” |
|  |  | *interprof* ACT intervention package: Shared goal setting (current practice) | Questions (e.g. „How often does it happen that decisions are being made about the medical care for your patients in the nursing home without involving you?”) to be answered by means of a 5-step rating scale ranging from 1=”very often” to 5=”never” |
|  |  | Key elements of interprofessional collaboration: Interprofessional communication (current practice) | Various questions regarding the ways and media used for RN-GP communication, categorial, ordinal and metric answer scales |
|  |  | Further domains related to interprofessional collaboration and medical care: General interprofessional collaboration (quality and satisfaction) | Questions (e.g. “Particular positive aspects of inter-professional collaboration with selected NH/GP”), open-ended (qualitative) |
|  |  | Context factors: Meso – organizational level: GP office and NH characteristics | Various questions regarding the characteristics of the GP offices and the NH, categorial and metric answer scales |
|  |  | Context factors: Micro – staff level: Expected effects of high quality RN-GP collaboration | Statements (e.g. “A well functioning cooperation between me, the general practitioner, and the nursing staff may reduce the frequency of hospital admissions of the nursing home residents.”) to be rated by means of a 4-step rating scale ranging from 1=“fully agree“ to 4=“do not agree“ |
|  |  | Context factors: Micro – staff level: Competences of RNs and GPs for medical care and interprofessional collaboration | Questions (e.g. “How satisfied you are with your own clinical competences”) to be rated by means of a 5-step rating scale ranging from 1=“absolutely“ to 5=“absolutely not“ |
|  |  | Context factors: Micro – NHR level: Family involvement in medical care | Questions (e.g. “How often does it happen that your relatives take care of your medical care, e.g. by arranging appointments with the general practitioner or general practitioner visits at your nursing home?“) to be answered by means of a 4-step rating scale ranging from 1= „regularly“ to 4=“can not say exactly“ |
| *Normalization Measure Development QUES (NoMAD)* [9, 10] | German [11] | Implementation strategies and activities: Implementational work within team: Coherence, cognitive participation, collective action, reflexive monitoring | Statements (e.g. “I will continue to support the implementation of the interprof ACT intervention package beyond the study.”) to be rated by means of a 5-step rating scale ranging from 1=absolutely agree to 5=absolutely not agree” |
| *Partnership Self-Assessment Tool (PSAT)* [12] | English* | *interprof* ACT intervention package: Shared goal setting (current practice, quality and satisfaction) | Questions (e.g. “How satisfied are you with the ways how are decisions being made in your collaboration with …?” to be rated by means of 5-step rating scale ranging from 1=“absolutely“ to 5=“absolutely not“ |
|  |  | Key elements of interprofessional collaboration: Contribution of involved professions (quality and satisfaction) | Questions (e.g. “How satisfied are you with your influence in the partnership?”) to be answered by means of a 5-step rating scale ranging from 1=“absolutely“ to 5=“absolutely not“ |
| *Practice Environment Scale of the Nursing Work Index (PES-NWI)* [13, 14] | German* [15] | Context factors: Meso – organizational level: Leadership and work environment | Statements (e.g. “High standards of nursing care are expected by the administration.”) to be answered by means of a 4-step rating scale ranging from 1=“fully agree“ to 4=“do not agree“ |
| *Zufriedenheit in der Arztpraxis (ZAP)* [16] | German | Key elements of interprofessional collaboration: Involvement of NHR (quality and satisfaction) | Questions (e.g. “How satisfied are you with the information given by your general practitioner about your health conditions and treatments?”) to be answered by means of a 4-step rating scale ranging from 1= “very satisfied“ to 4=“very dissatisfied“ |
|  |  | Further domains related to interprofessional collaboration and medical care: General (medical) care for NHR (quality and satisfaction) | Questions (e.g. “How often does your general practitioner explain the given information to you in an easily to understand way?” to be answered by a 4-step rating scale ranging from 1=“always“ to 4=“never“ |
|  |  | Context factors: Micro – NHR level: Utilisation of GP office (formal characteristics) | Questions (e.g. “For how long you have already visited your current general practitioner?” to be answered by 4 categorial answer options: “less than one year”, “1 to 2 years”, “3 to 5 years”, “more than 5 years” |

*Selected items were translated using forward and backward translation procedures. RN = Registered nurse. NHR = Nursing home residents. NH= Nursing home. GP = General practitioner.

**References:**

1. Schroder C, Medves J, Paterson M, Byrnes V, Chapman C, O'Riordan A, et al. Development and pilot testing of the collaborative practice assessment tool. J Interprof Care. 2010;25:189-95.
2. Klingenberg A, Bahrs O, Szecsenyi J. Wie beurteilen Patienten Hausärzte und ihre Praxen? Deutsche Ergebnisse der europäischen Studie zur Bewertung hausärztlicher Versorgung durch Patienten (EUROPEP). [How do patients evaluate general practice? German results from the European project on patient evaluation of general practice care (EUROPEP)]. Z Arztl Fortbild Qualitatssich. 1999;93:437-45.
3. Holle D, Halek M, Mayer H, Bartholomeyczik S. Die Auswirkungen der Verstehenden Diagnostik auf das Belastungserleben Pflegender im Umgang mit Menschen mit Demenz in der stationären Altenhilfe. [The influence of understanding diagnostics on perceived stress of nurses caring for nursing home residents with dementia]. Pflege. 2011;24:303-16
4. Hojat M, Fields SK, Veloski JJ, Griffiths M, Cohen MJ, Plumb JD. Psychometric properties of an attitude scale measuring physician-nurse collaboration. Eval Health Prof. 1999;22(2):208-20.
5. Hojat M, Nasca TJ, Cohen MJ, Fields SK, Rattner SL, Griffiths M, et al. Attitudes toward physician-nurse collaboration: a cross-cultural study of male and female physicians and nurses in the United States and Mexico. Nurs Res. 2001;50(2):123-8.
6. Hojat M, Gonnella JS, Nasca TJ, Fields SK, Cicchetti A, Lo Scalzo A, et al. Comparisons of American, Israeli, Italian and Mexican physicians and nurses on the total and factor scores of the Jefferson scale of attitudes toward physician-nurse collaborative relationships. Int J Nurs Stud. 2003;40(4):427-35.
7. Lakeit S. Nurse-Physican Collaboration. A systematic literature review measuring nurse-physican collaboration, and the development and psychometric testing of one of these instruments for a German Target Group. Dissertation. Köln: Universität zu Köln (University of Cologne); 2015.
8. van den Bussche H, Jahncke-Latteck ÄD, Ernst A, Tetzlaff B, Wiese B, Schramm U. Zufriedene hausärzte und kritische pflegende - probleme der interprofessionellen zusammenarbeit in der versorgung zu hause lebender menschen mit demenz. [Satisfied general practitioners and critical nursing staff - problems of interprofessional cooperation in the home care of dementia patients]. Gesundheitswesen. 2012;75:328-33.
9. Rapley T, Girling M, Mair FS, Murray E, Treweek S, McColl E, et al. Improving the normalization of complex interventions: part 1 - development of the NoMAD instrument for assessing implementation work based on normalization process theory (NPT). BMC Med Res Methodol. 2018;18:133.
10. Finch TL, Girling M, May CR, Mair FS, Murray E, Treweek S, et al. Improving the normalization of complex interventions: part 2 - validation of the NoMAD instrument for assessing implementation work based on normalization process theory (NPT). BMC Med Res Methodol. 2018;18:135.
11. ImpleMentAll Consortium. Normalization Measure development questionnaire (short NOMAD). German Version. 2018. [https://www.implementall.eu/NoMAD_German.pdf. Accessed 25 Nov 2021](https://www.implementall.eu/NoMAD_German.pdf.%20Accessed%2025%20Nov%202021).
12. Center for the Advancement of Collaborative Strategies in Health. Partnership self-assessment tool questionnaire. 2002. https://atrium.lib.uoguelph.ca/xmlui/bitstream/handle/10214/3129/Partnership_Self-Assessment_Tool-Questionnaire_complete.pdf?sequence=1&isAllowed=y. Accessed 12 Dec 2021.
13. Aiken LH, Patrician PA. Measuring organizational traits of hospitals: the revised nursing work index. Nurs Res. 2000;49:146-53.
14. Lake ET. Development of the practice environment scale of the nursing work index. Res Nurs Health. 2002;25:176-88.
15. Zander B, Dobler L, Busse R. The introduction of DRG funding and hospital nurses' changing perceptions of their practice environment, quality of care and satisfaction: comparison of cross-sectional surveys over a 10-year period. Int J Nurs Stud. 2013;50(2):219-29.
16. Bitzer EM, Dierks ML, Dörning H, Schwartz FW. Zufriedenheit in der arztpraxis aus patientenperspektive – psychometrische prüfung eines standardisierten erhebungsinstrumentes. [Patient satisfaction with ambulatory care physicians - psychometric testing of a standardized questionnaire]. Z Gesundh Wiss. 1999;7:196-209.
